# Risk factors and predictive models of poor prognosis and delayed cerebral ischemia in aneurysmal subarachnoid hemorrhage complicated with hydrocephalus

**DOI:** 10.3389/fneur.2022.1014501

**Published:** 2022-10-24

**Authors:** Lintao Wang, Qingqing Zhang, Gaoqi Zhang, Wanwan Zhang, Wenwu Chen, Fandi Hou, Zhanqiang Zheng, Yong Guo, Zhongcan Chen, Yanxia Wang, Juha Hernesniemi, Hugo Andrade-Barazarte, Xiaohui Li, Tianxiao Li, Guang Feng, Jianjun Gu

**Affiliations:** ^1^Department of Neurology, The First Affiliated Hospital of Henan University, Kaifeng, China; ^2^School of Clinical Medicine, Henan University, Kaifeng, China; ^3^Department of Neurosurgery, Henan University People's Hospital, Henan Provincial People's Hospital, Zhengzhou, China; ^4^Department of Neurosurgery, Zhengzhou University People's Hospital, Henan Provincial People's Hospital, Zhengzhou, China

**Keywords:** aneurysmal subarachnoid hemorrhage (aSAH), hydrocephalus, inflammation, Hunt-Hess grade, delayed cerebral ischemia (DCI), prognosis, outcome, risk factor

## Abstract

**Objective:**

To evaluate the correlation of serum biological markers and related scales to the occurrence of delayed cerebral ischemia and clinical prognosis in patients with aneurysmal subarachnoid hemorrhage (aSAH) complicated with acute hydrocephalus before admission.

**Methods:**

The clinical data of 227 patients with pre-admission aSAH complicated with acute hydrocephalus admitted to Henan Provincial People's Hospital from April 2017 to December 2020 were retrospectively analyzed. Patients were grouped according to the presence or absence of delayed cerebral ischemia (DCI) after surgery and the prognosis at 6 months after discharge. Univariate and multivariable logistic regression analysis were performed to analyze the relationship between serum biological indicators combined with aneurysm related clinical score scale and the occurrence and prognosis of delayed cerebral ischemia. ROC curves and nomogram were drawn.

**Results:**

Multivariable Logistic regression analysis showed that high Hunt-Hess grade and surgical clipping were independent risk factors for postoperative DCI (*P* < 0.05). Older age, higher Hunt-Hess grade, higher CRP and neutrophil levels were independent risk factors for poor prognosis at 6 months after surgery (*P* < 0.05). ROC curve analysis showed that the area under the curve (AUC) of Hunt-Hess grade and surgical method for predicting DCI in patients with aSAH combined with hydrocephalus after surgery were 0.665 and 0.593. The combined AUC of Hunt-Hess grade and surgical method was 0.685, the sensitivity was 64.9%, and the specificity was 64.7%. The AUC of CRP, neutrophil, age and Hunt-Hess grade for predicting poor prognosis in patients with aSAH combined with hydrocephalus at 6 months after surgery were 0.804, 0.735, 0.596, 0.757, respectively. The combined AUC of CRP, neutrophil, age, Hunt-Hess grade was 0.879, the sensitivity was 79%, and the specificity was 84.5%. According to the correction curve, the predicted probability of the nomogram is basically consistent with the actual probability.

**Conclusion:**

Hunt-Hess grade and surgical method are independent predictors of postoperative DCI in patients with aSAH complicated with hydrocephalus. “CRP,” “neutrophil,” “age” and “Hunt-Hess grade” at admission are independent predictors of clinical prognosis in patients with aSAH complicated with hydrocephalus. The combination of the above indicators has high predictive value.

## Introduction

Aneurysmal subarachnoid hemorrhage (aSAH) is one of the most common neurological emergencies in clinical practice, accounting for about 5% of stroke patients (1, 2), with a mortality rate of 35%, and about one third of survivors are left with permanent disability (3, 4). Delayed cerebral ischemia is one of the main causes of high mortality and morbidity (5). DCI is a clinical imaging syndrome that includes focal ischemia and/or cognitive impairment on CT/MRI and/or cerebral infarction (6).

Hydrocephalus is a common serious complication of aSAH, and about 20 to 30% of aSAH patients will develop acute hydrocephalus due to obstruction of cerebrospinal fluid circulation or abnormal absorption (7–9). Acute hydrocephalus will not only aggravate the early neurological impairment of patients with aSAH, further aggravate the clinical condition, but also impair the neurological function of patients in the recovery period after aSAH surgery (10). Although patients with aSAH with acute hydrocephalus can be treated with surgery or lumbar cisternae drainage, these patients represent a subgroup of patients with more advanced aSAH. Therefore, for patients with aSAH complicated with hydrocephalus on admission, searching for biomarkers that can predict the prognosis of the patients is conducive to early formulation of a reasonable treatment plan, so as to improve the prognosis and reduce the mortality of the patients.

In recent years, more and more attention has been paid to the determination of various biomarkers in blood. Studies have shown that Inflammatory markers, blood cell markers, blood glucose markers, blood lipid markers, and aneurysmal-related clinical scoring scales (such as Hunt-Hess scale, modified Fisher scale, and WFNS scale) have high clinical reference value in predicting the clinical prognosis of patients without hydrocephalus and aSAH at admission (11–16). However, no studies have shown the prognostic value of clinical blood indicators or other related indicators in patients with aSAH complicated with acute hydrocephaluson admission.

The purpose of this study was to retrospectively analyze the clinical data of aSAH patients with acute hydrocephalus at admission and the prognostic results at 6 months after surgery, and to explore the predictive value of blood biomarkers and aneurysmal-related clinical score scale for the presence of DCI and different prognosis in aSAH patients with acute hydrocephalus after surgery. It provides sufficient theoretical basis for early clinical intervention and improvement of prognosis of aSAH patients.

## Materials and methods

### General information

A total of 227 patients with aSAH complicated with hydrocephalus admitted to Henan Provincial People's Hospital from April 2017 to December 2020 were retrospectively enrolled. One hundred and fifty were females and 77 were males.

The inclusion criteria were as follows: (1) aSAH confirmed by head CT or CT angiography (CTA) or DSA; (2) Hydrocephalus was found by head CT examination on the day of admission; (3) Intervention or craniotomy clipping treatment within 10 days after admission; (4) 18–80 years old; 5. Complete admission and venous blood collection within 24 h after onset; (6) Come to our hospital for review 6 months after operation; (7) No new neurological diseases occurred 6 months after discharge; (8) CT findings of new infarction in DCI patients are not related to surgery.

The exclusion criteria were as follows: (1) non-aneurysmal subarachnoid hemorrhage, such as moyamoya disease, arteriovenous malformations, etc.; (2) History of hydrocephalus; (3) Sequelae of previous neurological diseases; (4) Suffering from serious systemic diseases, such as heart, liver, renal insufficiency, and blood system diseases; (5) Complicated with other intracranial diseases, such as intracranial infection and intracranial artery stenosis; (6) A history of taking aspirin, non-steroidal drugs, anti-inflammatory and analgesic drugs 6 months before admission; (7) Pregnant or lactating women; (8) Incomplete clinical data.

### Methods

The basic clinical data of the patients were collected, including age, gender, smoking history, drinking history, underlying diseases (hypertension, coronary heart disease, diabetes mellitus, cerebral infarction), Hunt-Hess grade on admission, imaging examination data, aneurysm location record, modified Fisher grade, etc. Hematological indexes (inflammatory indexes, blood cell indexes, blood lipid indexes) were recorded within 24 h after admission. The patients were divided into DCI group and non-DCI group according to whether DCI occurred after operation. The patients were divided into good prognosis group (mRS ≤2) and poor prognosis group (mRS >2) by modified Rankin score (mRS) according to the condition at 6 months after operation.

### Statistical analysis

SPSS 25.0 statistical software was used for data processing. Measurement data conforming to normal distribution were expressed as ($\bar{x}$ ± s), and comparison between groups was performed by *t*-test. Measurement data that did not conform to normal distribution were also expressed as ($\bar{x\;}$ ± s), and comparison between groups was performed by Mann-Whitney U-test. Statistical data were expressed as percentage (%), and comparison between groups was performed by χ2 test. Factors that might predict prognosis were used as independent variables in univariate analysis. On the basis of univariate analysis, indicators with *P* < 0.05 were included in multivariable Logistic regression analysis to generate independent risk factors. Receiver Operating characteristic curve (ROC) was established to determine the AUC, sensitivity, specificity and cutoff values of the final prognostic indicators. ROC curve was drawn by combining the significant indexes in multivariable analysis. Based on the results of multivariable logistic regression, the Nomogram was drawn using R4.2 software for meaningful indicators, and 1,000 Bootstrap samples were corrected to measure the predictive ability of Nomogram and reduce overfitting bias. In this study, *P* < 0.05 was defined as statistically significant.

## Results

**Figure 1:**
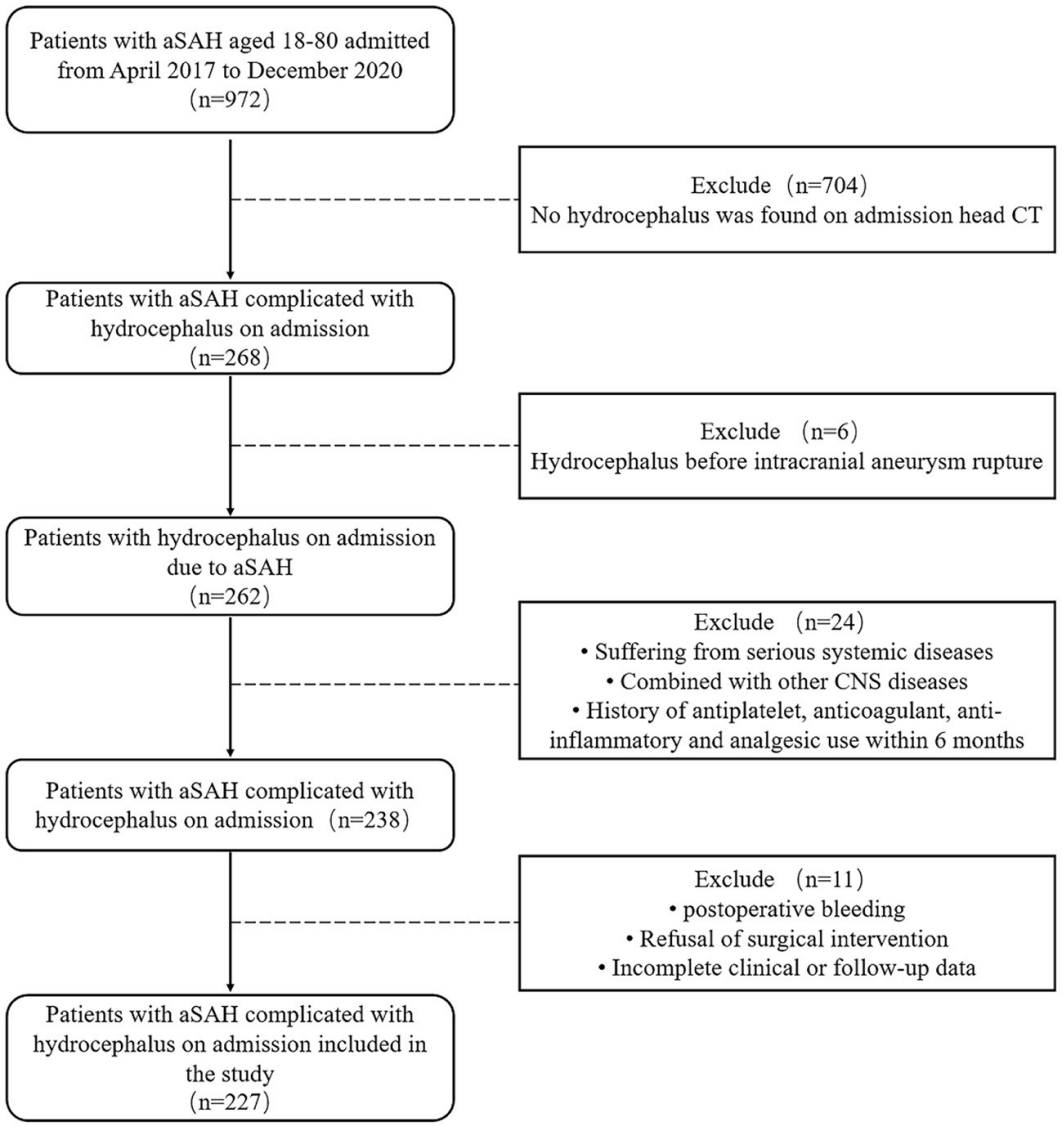
Flow chart of patient selection.

A total of 227 patients with aSAH complicated with hydrocephalus were enrolled (Figure 1). According to the occurrence of postoperative DCI, they were divided into DCI group (74 cases) and non-DCI group (153 cases). There were 44 females and 30 males in DCI group, with an average age of (65.16 ± 9.85) years. There were 106 females and 47 males in the non-DCI group, with an average age of (63.36 ± 11.38) years. In DCI group, 55 ruptured aneurysms were located in anterior circulation and 19 ruptured aneurysms were located in posterior circulation. Thirty seven cases received interventional therapy and 37 cases received clipping therapy. In the non-DCI group, 94 ruptured aneurysms were located in the anterior circulation and 59 in the posterior circulation. One hundred and five cases were treated with interventional therapy and 48 cases were treated with clipping therapy (Table 1).

**Table 1:** Univariate analysis (Based on DCI group, non-DCI group).

**Characteristics**	**Non-DCI (*n* = 153)**	**DCI (*n* = 74)**	***P*-value**
**Gender**			*P* = 0.143
Female	106 (69.28%)	44 (59.46%)	
Male	47 (30.72%)	30 (40.54%)	
Age (years)	63.36 ± 11.38	65.16 ± 9.85	*P* = 0.277
Smoking history	23 (15.03%)	17 (22.97%)	*P* = 0.141
Drinking history	21 (13.73%)	8 (10.81%)	*P* = 0.537
Diabetes	15 (9.80%)	5 (6.76%)	*P* = 0.448
Hypertension	96 (62.75%)	48 (64.86%)	*P* = 0.756
Cerebral infarction	19 (12.42%)	12 (16.22%)	*P* = 0.411
Coronary heart disease	15 (9.80%)	14 (18.92%)	*P* = 0.054
**Modified Fisher grade**			*P* = 0.005
Grade I	13 (8.50%)	2 (2.70%)	
Grade II	81 (52.94%)	30 (40.54%)	
Grade III	26 (16.99%)	16 (21.62%)	
Grade IV	33 (21.57%)	26 (35.14%)	
**Hunt and Hess grade**			*P* = 0.000
Grade I	1 (0.65%)	0	
Grade II	55 (35.95%)	8 (10.81%)	
Grade III	62 (40.52%)	35 (47.30%)	
Grade IV	27 (17.65%)	21 (28.38%)	
Grade V	8 (5.23%)	10 (13.51%)	
**Surgical method**			*P* = 0.007
Interventional	105 (68.63%)	37 (50.00%)	
Clipping	48 (31.37%)	37 (50.00%)	
**Location of aneurysm**			*P* = 0.055
Anterior circulation	94 (61.44%)	55 (74.32%)	
Posterior circulation	59 (38.56%)	19 (25.68%)	
**Laboratory results**			
CRP (mg/L)	22.21 ± 33.19	33.67 ± 36.93	*P* = 0.002
WBC (×10^9^/L)	10.99 ± 4.52	13.16 ± 4.50	*P* = 0.001
Neutrophil (×10^9^/L)	9.24 ± 4.39	11.20 ± 4.00	*P* = 0.000
Lymphocyte (×10^9^/L)	1.04 ± 0.50	1.01 ± 0.49	*P* = 0.699
Monocyte (×10^9^/L)	0.67 ± 1.18	0.64 ± 0.34	*P* = 0.178
Eosinophil (×10^9^/L)	0.05 ± 0.16	0.02 ± 0.06	*P* = 0.027
Procalcitonin (ng/mL)	0.21 ± 0.06	0.23 ± 0.06	*P* = 0.094
Hematocrit	0.37 ± 0.05	0.38 ± 0.37	*P* = 0.098
RDW-CV (%)	12.85 ± 1.18	13.05 ± 1.19	*P* = 0.376
MPV (fL)	10.32 ± 1.37	14.85 ± 36.82	*P* = 0.113
PLT (×10^9^/L)	213.90 ± 65.75	214.84 ± 74.81	*P* = 0.787
P-LCR (%)	28.53 ± 9.64	29.93 ± 9.99	*P* = 0.396
D-Di (μg/ml)	3.63 ± 8.01	3.61 ± 3.84	*P* = 0.002
TC (mmol/L)	4.58 ± 1.07	4.44 ± 1.10	*P* = 0.392
TG (mmol/L)	1.74 ± 1.74	1.38 ± 0.80	*P* = 0.380
HDL (mmol/L)	1.24 ± 0.34	1.22 ± 0.33	*P* = 0.720
LDL (mmol/L)	2.60 ± 0.83	2.51 ± 0.81	*P* = 0.438
Apo A1 (g/L)	1.13 ± 0.29	1.06 ± 0.27	*P* = 0.097
Apo B100 (g/L)	0.85 ± 0.23	0.84 ± 0.24	*P* = 0.783
FBG (g/L)	3.76 ± 1.30	3.88 ± 1.13	*P* = 0.289
ALB (g/L)	38.27 ± 5.49	39.41 ± 5.00	*P* = 0.197

CRP, C reactive protein; WBC, white blood cell; RDW-CV, red blood cell distribution width-CV; MPV, mean platelet volume; PLT, platelet; P-LCR, platelet-large cell ratio; D-Di, D-Dimer; TC, total cholesterol; TG, triglyceride; HDL, high density lipoprotein; LDL, low density lipoprotein; Apo A1, apolipoprotein a1; Apo B100 = apolipoprotein B100; FBG, fibrinogen; ALB, albumin.

According to mRS score at 6 months after surgery, the patients were divided into good prognosis group (103 cases) and poor prognosis group (124 cases). In the good prognosis group, there were 74 females and 29 males, with an average age of (61.96 ± 11.13) years. There were 76 females and 48 males in the poor prognosis group, with an average age of (65.60 ± 10.49) years. In the good prognosis group, 70 ruptured aneurysms were in the anterior circulation and 33 ruptured aneurysms were in the posterior circulation. Seventy one cases were treated with interventional therapy and 32 cases were treated with clipping therapy. In the poor outcome group, 79 ruptured aneurysms were in the anterior circulation and 45 were in the posterior circulation. Seventy one cases received interventional therapy and 53 cases received clipping therapy (Table 2).

**Table 2:** Univariate analysis (Based on good prognosis group, poor prognosis group).

**Characteristics**	**Good outcome** (**mRS 0–2, *n* = 103)**	**Poor outcome** **(mRS 3–6, *n* = 124)**	***P*-value**
**Gender**			*P =* 0.094
Female	74 (71.84%)	76 (61.29%)	
Male	29 (28.16%)	48 (38.71%)	
**Age (years)**	61.96 ± 11.13	65.60 ± 10.49	*P =* 0.013
Smoking history	16 (15.53%)	24 (19.35%)	*P =* 0.452
Drinking history	13 (12.62%)	16 (12.90%)	*P =* 0.949
Diabetes	10 (9.71%)	10 (8.06%)	*P =* 0.663
Hypertension	65 (63.11%)	79 (63.71%)	*P =* 0.925
Cerebral infarction	13 (12.62%)	19 (15.32%)	*P =* 0.661
Coronary heart disease	13 (12.62%)	16 (12.90%)	*P =* 0.949
**Modified Fisher grade**			*P =* 0.000
Grade I	12 (11.65%)	3 (2.42%)	
Grade II	64 (62.14%)	47 (37.90%)	
Grade III	16 (15.53%)	26 (20.97%)	
Grade IV	11 (10.68%)	48 (38.71)	
**Hunt and Hess grade**			*P =* 0.000
Grade I	1 (0.97%)	0	
Grade II	46 (44.66%)	17 (13.71%)	
Grade III	48 (46.60%)	49 (39.52%)	
Grade IV	7 (6.80%)	41 (33.06%)	
Grade V	1 (0.97%)	17 (13.71%)	
**Surgical method**			*P =* 0.070
Interventional	71 (68.93%)	71 (57.26%)	
Clipping	32 (31.07%)	53 (42.74%)	
**Location of aneurysm**			*P =* 0.502
Anterior circulation	70 (67.96%)	79 (63.71%)	
Posterior circulation	33 (32.04%)	45 (36.29%)	
**Laboratory results**			
CRP (mg/L)	9.85 ± 12.86	39.32 ± 41.11	*P =* 0.000
WBC (×10^9^/L)	10.24 ± 3.69	12.90 ± 4.96	*P =* 0.000
Neutrophil (×10^9^/L)	8.06 ± 3.19	11.38 ± 4.63	*P =* 0.000
lymphocyte (×10^9^/L)	1.11 ± 0.49	0.96 ± 0.49	*P =* 0.011
Monocyte (×10^9^/L)	0.73 ± 1.42	0.60 ± 0.34	*P =* 0.960
Eosinophil (×10^9^/L)	0.05 ± 0.11	0.03 ± 0.16	*P =* 0.001
Procalcitonin (ng/mL)	0.22 ± 0.06	0.22 ± 0.07	*P =* 0.664
Hematocrit	0.37 ± 0.04	0.38 ± 0.05	*P =* 0.624
RDW-CV (%)	13.02 ± 1.23	12.83 ± 1.14	*P =* 0.301
MPV (fL)	10.38 ± 1.38	12.97 ± 28.47	*P =* 0.483
PLT (×10^9^/L)	214.72 ± 60.60	213.78 ± 74.95	*P =* 0.793
P-LCR (%)	29.11 ± 10.60	28.91 ± 9.11	*P =* 0.897
D-Di (μg/ml)	1.93 ± 4.60	5.02 ± 8.13	*P =* 0.000
TC (mmol/L)	4.60 ± 0.99	4.47 ± 1.15	*P =* 0.420
TG (mmol/L)	1.66 ± 1.79	1.57 ± 1.21	*P =* 0.418
HDL (mmol/L)	1.27 ± 0.30	1.21 ± 0.35	*P =* 0.158
LDL (mmol/L)	2.61 ± 0.76	2.53 ± 0.86	*P =* 0.496
Apo A1 (g/L)	1.14 ± 0.21	1.08 ± 0.32	*P =* 0.106
Apo B100 (g/L)	0.84 ± 0.21	0.84 ± 0.24	*P =* 0.935
FBG (g/L)	3.67 ± 1.17	3.91 ± 1.30	*P =* 0.165
ALB (g/L)	38.99 ± 5.64	38.42 ± 5.06	*P =* 0.504

### Univariate analysis

There were no significant differences in DCI group with non-DCI in gender, age, smoking history, drinking history, diabetes, hypertension, cerebral infarction, coronary heart disease, aneurysm location, lymphocyte, monocyte, procalcitonin, hematocrit, red blood cell distribution width, average blood platelet volume, platelet count, large platelet volume, total cholesterol, triglyceride, high density lipoprotein, low density lipoprotein, apolipoprotein A1, apolipoprotein B100, fibrinogen and albumin levels (*P* > 0.05). The levels of Hunt-Hess grade, modified Fisher grade, CRP, white blood cells, neutrophil and D-dimer in DCI group were higher than those in non-DCI group (*P* < 0.05). The amount of surgical clipping in the DCI group was higher than that in the non-DCI group (*P* < 0.05). The level of eosinophil in DCI group was lower than that in non-DCI group (*P* < 0.05) (Table 1).

There were no significant differences in good prognosis group with poor prognosis in gender, smoking history, drinking history, diabetes, hypertension, cerebral infarction, coronary heart disease, surgical procedure, aneurysm location, monocyte, procalcitonin, hematocrit, red blood cell distribution width, average blood platelet volume, platelet count, large platelet volume, total cholesterol, triglyceride, high density lipoprotein, low density lipoprotein, apolipoprotein A1, apolipoprotein B100, fibrinogen and albumin levels (*P* > 0.05). The age, Hunt-Hess grade, modified Fisher grade, CRP, leukocyte, neutrophil, lymphocyte, eosinophil and D-dimer levels in the poor prognosis group were higher than those in the good prognosis group, with statistical significance (*P* < 0.05) (Table 2).

### Multivariable logistic regression analysis

According to the results of univariate analysis, the occurrence of DCI was taken as the dependent variable. Hunt-Hess grade, modified Fisher grade, surgical approach (intervention, clipping), CRP, leukocytes, neutrophils, eosinophils, and D-dimer levels were used as independent variables. Logistic regression model analysis showed that “Hunt-Hess grade was high” (OR = 1.900, 95%Cl: 1.359–2.657) and “surgical clipping” (OR = 2.069, 95%Cl: 1.137–3.765) increased the risk of DCI compared with intervention. Hunt-Hess grade and surgical clipping were independent risk factors for postoperative DCI in patients with aSAH complicated with hydrocephalus (*P* < 0.05) (Table 3).

**Table 3:** Multivariable logistic analysis (Based on DCI group, non-DCI group).

**Characteristics**	**OR**	**95% CI**	***P*-value**
Hunt-Hess grade	1.900	1.359–2.657	0.000
**Surgical method**			
Interventional	1		
Clipping	2.069	1.137–3.765	0.017
Modified Fisher grade			0.318
CRP			0.199
WBC			0.059
Neutrophil			0.234
Eosinophil			0.256
D-Di			0.777

OR, odds ratio; Cl, confidence interval.

According to the results of univariate analysis, the prognosis at 6 months after surgery (good and poor) was used as the dependent variable. Age, Hunt-Hess grade, modified Fisher grade, CRP, leukocytes, neutrophils, lymphocytes, eosinophils and D-dimer were used as independent variables. Logistic regression model analysis showed that the independent risk factors for poor prognosis in patients with aSAH complicated with hydrocephalus at 6 months after surgery were “increased age” (OR = 1.038, 95%Cl: 1.003–1.074) and “increased CRP” (OR = 1.057, 95%Cl: 1.031–1.084), “increased neutrophils” (OR = 1.212, 95%Cl: 1.076–1.365) and “high Hunt-Hess grade” (OR = 2.538, 95%Cl: 1.532–4.208) (*P* < 0.05) (Table 4).

**Table 4:** Multivariable logistic analysis (Based on good prognosis group, poor prognosis group).

**Characteristics**	**OR**	**95% CI**	***P*-value**
Age	1.038	1.003–1.074	0.033
CRP	1.057	1.031–1.084	0.000
Neutrophil	1.212	1.076–1.365	0.002
Hunt-Hess grade	2.538	1.532–4.208	0.000
Modified Fisher grade			0.103
WBC			0.074
Lymphocyte			0.922
Eosinophil			0.777
D-Di			0.064

OR, odds ratio; Cl, confidence interval.

### Nomogram

**Figure 2:**
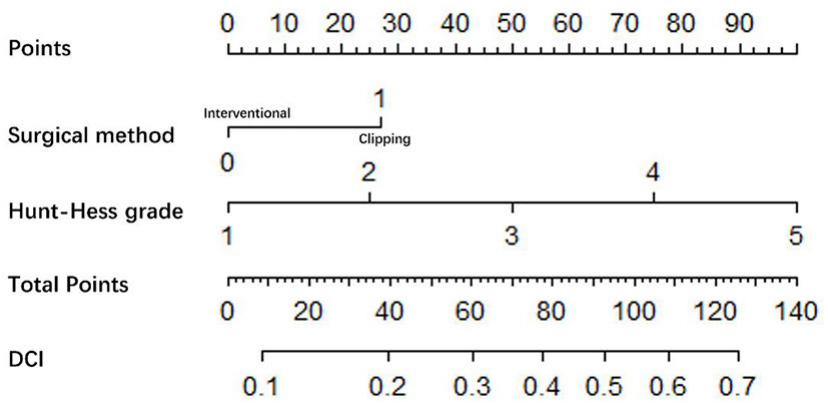
Nomogram (Based on DCI group, non-DCI group).

**Figure 3:**
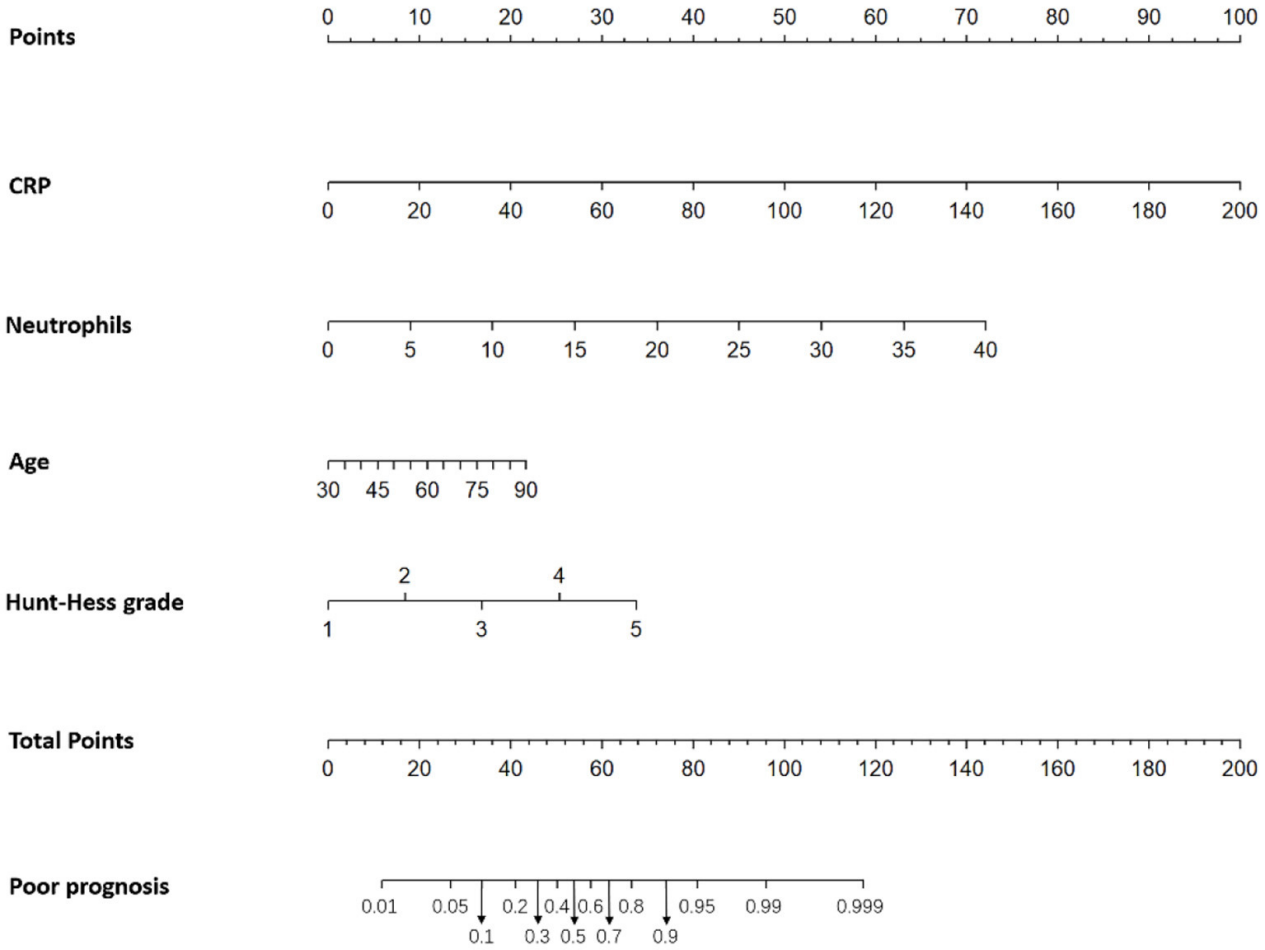
Nomogram (Based on good prognosis group, poor prognosis group).

Significant logistic analysis indicators were used to construct Nomogram (Figures 2, 3). Nomogram can be used to assess the risk of DCI and poor prognosis in patients with aSAH complicated with acute hydrocephalus.

The probability of DCI was predicted according to the risk value corresponding to the total score, which was calculated according to the patient's “Hunt-Hess grade” and “surgical method.” And the contribution of each factor to the overall risk of DCI was revealed (Figure 2).

The probability of poor prognosis was predicted according to the risk value corresponding to the total score, which was calculated according to the patient's “age,” “CRP,” “neutrophil” and “Hunt-Hess grade.” And the contribution of each factor to the overall risk of poor prognosis was revealed. For example, for a “72-year-old” aSAH patient with acute hydrocephalus, “CRP = 25.47 mg/L,” “neutrophil = 9.4 × 10^9^/L,” “Hunt-Hess grade 4,” the probability of poor prognosis is 84%. According to the positions of each factor of the Nomogram, 15 points of “72 years old,” 13 points of “CRP = 25.47 mg/L,” 17 points of “neutrophil = 9.4 × 10^9^/L,” and 25 points of “Hunt-Hess grade 4” were calculated. Then the positions of each factor were summed to obtain a total score of 70. A score of 70 corresponds to an ~84% probability of poor prognosis (Figure 3).

**Figure 4:**
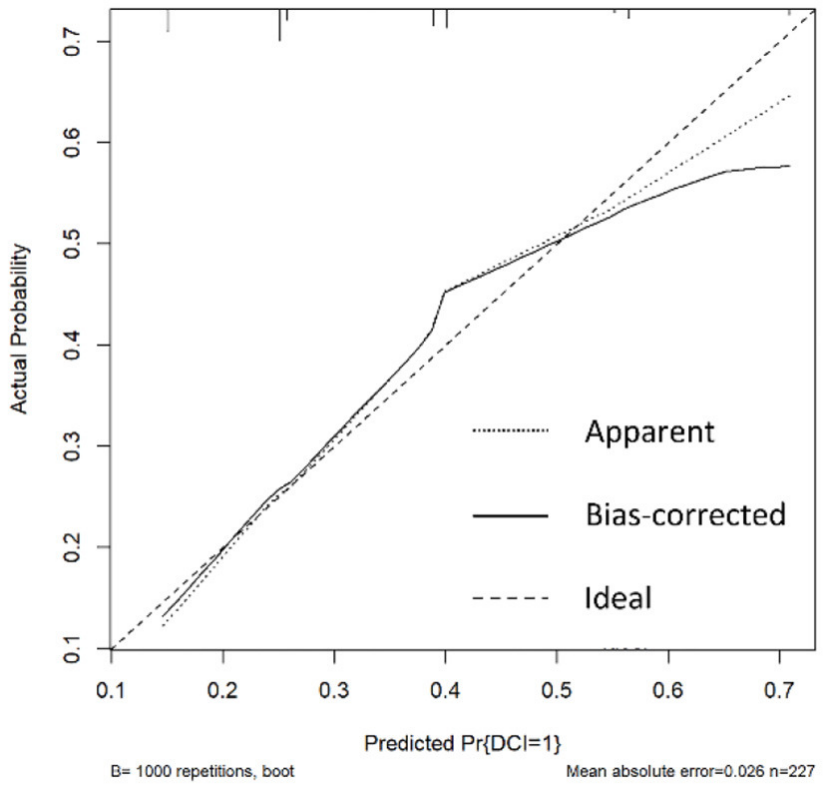
Calibration curve (Based on DCI group, non-DCI group).

**Figure 5:**
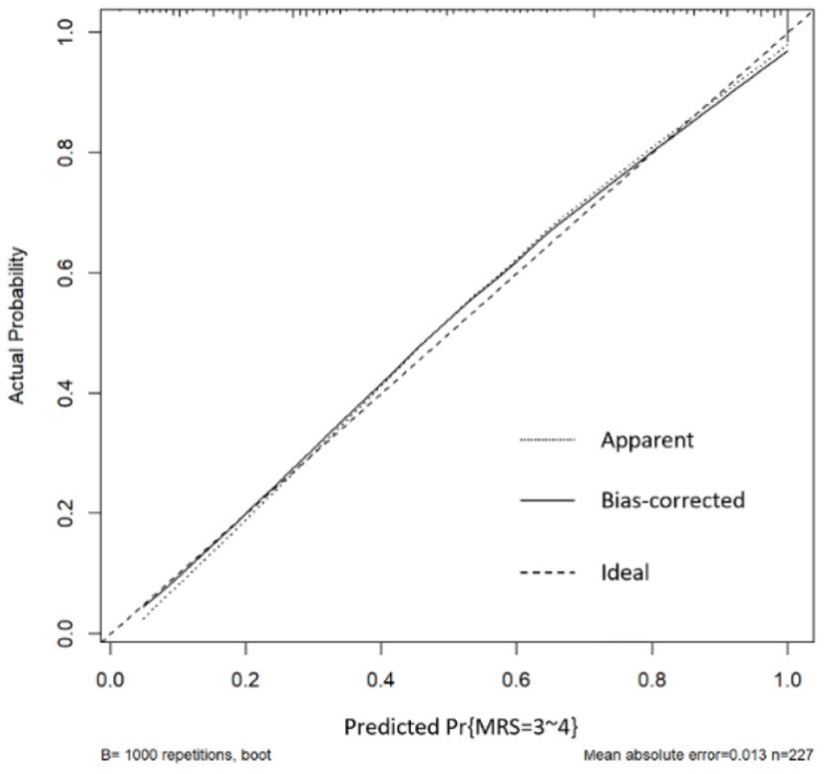
Calibration curve (Based on good prognosis group, poor prognosis group).

Verify the Nomogram: The calibration curve of the prediction model with 1,000 duplicate samples established by bootstrapping method has high accuracy (Figures 4, 5).

### ROC curve analysis

**Figure 6:**
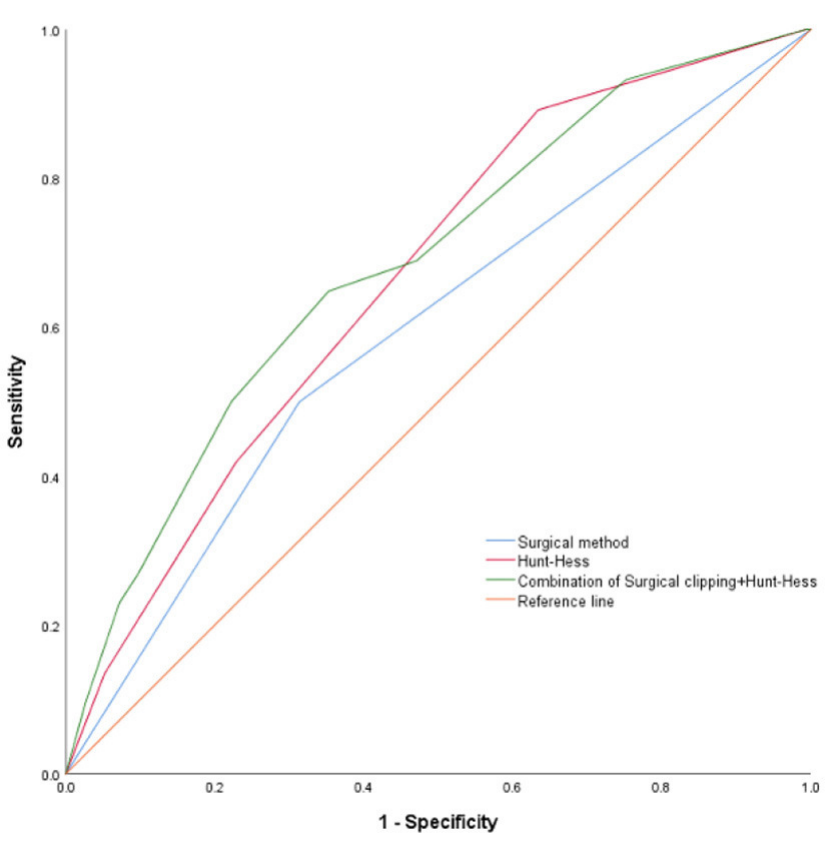
ROC curve (Based on DCI group, non-DCI group).

The optimal cut-off values of “Hunt-Hess grade” was 2.5. The AUC of “Hunt-Hess grade” and “surgical method” were 0.665 and 0.593, respectively, in predicting the DCI of aSAH combined with hydrocephalus after surgery. The sensitivities were 89.2 and 50%, respectively. The specificity ware 36.6 and 68.6%, respectively. The AUC of “ Hunt-Hess grade + surgical method” was 0.685, the sensitivity was 64.9%, and the specificity was 64.7% (Table 5 and Figure 6).

**Table 5:** The AUC, cut-off value, sensitivity, specificity from the ROC curve (Based on DCI group, non-DCI group).

**Characteristics**	**AUC**	**95% CI**	**Cut-off**	**Sensitivity (%)**	**Specificity (%)**
Hunt-Hess grade	0.665	0.592–0.737	2.5	89.2	36.6
Surgical method	0.593	0.513–0.673	-	50	68.6
Combination	0.685	0.611–0.758	-	64.9	64.7

**Figure 7:**
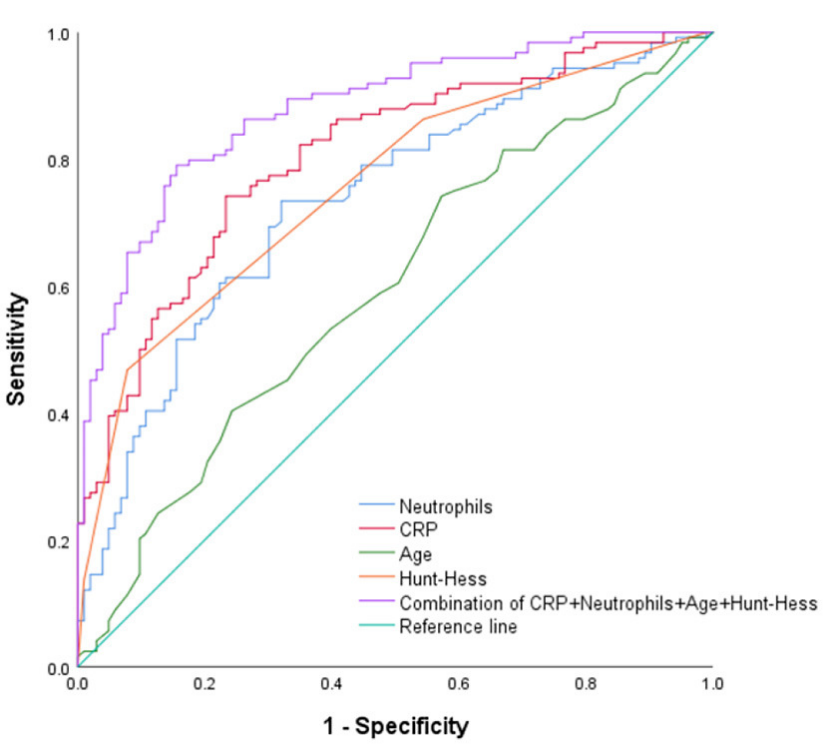
ROC curve (Based on good prognosis group, poor prognosis group).

The optimal cut-off values of “age,” “Hunt-Hess grade,” “CRP” and “neutrophil” were 61.5, 3.5, 11.4, and 9.06, respectively, in predicting the poor prognosis of aSAH combined with hydrocephalus at 6 months after surgery. The AUC were 0.596, 0.757, 0.804, and 0.735, respectively. The sensitivities were 74.2, 46.8, 74.2, and 73.4%, respectively. The specificity ware 42.7, 92.2, 76.7, and 68%, respectively. The AUC of “age + Hunt-Hess grade + CRP + neutrophils” was 0.879, the sensitivity was 79%, and the specificity was 84.5% (Table 6 and Figure 7).

**Table 6:** The AUC, cut-off value, sensitivity, specificity from the ROC curve (Based on good prognosis group, poor prognosis group).

**Characteristics**	**AUC**	**95% CI**	**Cut-off**	**Sensitivity (%)**	**Specificity (%)**
CRP	0.804	0.748–0.861	11.4	74.2	76.7
Neutrophil	0.735	0.670–0.800	9.06	73.4	68
Age	0.596	0.522–0.670	61.5	74.2	42.7
Hunt-Hess grade	0.757	0.695–0.819	3.5	46.8	92.2
Combination	0.879	0.835–0.923	-	79	84.5

## Discussion

Aneurysmal subarachnoid hemorrhage often causes hydrocephalus (17, 18), which can aggravate neurological impairment and seriously affect the prognosis of patients. Patients with aSAH complicated with acute hydrocephalus have a poor prognosis and high mortality, and even those who survive are often left with neurological damage or cognitive impairment (10). aSAH-associated hydrocephalus is thought to occur through the following mechanisms: (1) Blood products hinder the normal circulation of cerebrospinal fluid; (2) Excessive secretion of cerebrospinal fluid; (3) Cerebrospinal fluid reabsorption is reduced due to blood products affecting arachnoid granules (19–21). In addition, the subarachnoid inflammatory response to blood products may vary depending on the severity of the patient. From the perspective of the physiological mechanism of acute hydrocephalus after aSAH, the degree of inflammatory response of subarachnoid space to blood products determines the effect of cerebrospinal fluid obstruction and cerebrospinal fluid overproduction on acute hydrocephalus. Thus, this group of patients with early post-aSAH hydrocephalus, that is, acute hydrocephalus before aSAH admission, may represent a subgroup of patients with a stronger inflammatory response. Therefore, it is of great clinical significance to study the predictors of postoperative DCI and poor prognosis in patients with aSAH complicated with acute hydrocephalus, and to take targeted preventive measures as early as possible for high-risk patients with poor prognosis, which is helpful to improve the prognosis of patients.

### Delayed cerebral ischemia

DCI will seriously affect the prognosis of aSAH patients (6). Consistent with previous studies, the presence of DCI in this study was associated with poor prognosis (14.56% of patients with good prognosis had DCI, and 47.58% of patients with poor prognosis had DCI, *P* < 0.05).

In recent years, with the continuous development of imaging equipment and minimally invasive techniques, and the use of drugs to prevent cerebral vasospasm, the incidence of DCI after intracranial aneurysm surgery has been reduced (4). However, the incidence of DCI after aneurysm clipping and interventional therapy remains controversial. Ding et al. (5) believed that there was no significant difference in the incidence of DCI between clipping and interventional therapy. Dorhout et al. (22) believe that compared with interventional surgery, clipping is more likely to lead to DCI. Meta-analysis by Oliveira et al. (23) showed that there was no statistical difference in the incidence of postoperative DCI between the clipping group and the intervention group. Compared with interventional surgery, clipping surgery can remove the thrombus, but it also causes some damage to the vessel wall, thus increasing the incidence of DCI. This study confirmed that the incidence of DCI was higher with clipping than with intervention, which may be due to more severe brain injury with clipping than with intervention. Therefore, it is important to understand the differences of postoperative DCI between different surgical methods and effectively choose different surgical methods according to the patient's condition to reduce the incidence of postoperative DCI.

### Inflammatory indicators

Inflammation runs through the whole process of aSAH injury mechanism. When aneurysms rupture, blood cells deposited in the subarachnoid space stimulate brain tissue and activate immune regulatory cells in the central nervous system, and a large number of inflammatory cells enter the subarachnoid space, rapidly causing an inflammatory response (24, 25). On the other hand, in patients with aSAH combined with hydrocephalus, cerebrospinal fluid circulation pathway is impaired and CSF secretion/absorption is unbalanced, which makes inflammatory cells accumulate in the subarachnoid space and further aggravate the inflammatory response (24, 26–28). Inflammatory reaction can activate the immune system to release inflammatory cells and inflammatory mediators, leading to the occurrence and development of cerebral vasospasm, aggravating the occurrence of brain injury, and affecting the prognosis of patients (25, 29).

Inflammatory signal transduction pathway plays an important role in early neuronal injury after SAH. Heme, oxygenated hemoglobin, methemoglobin, peroxidase-2, matricellular proteins, heat shock protein, fibrinogen and so on produced by erythrocyte degradation after aneurysm rupture can lead to the activation of early inflammatory signaling pathways (such as TLR4) in aSAH when large amounts of blood enter the subarachnoid space (28, 30). On the one hand, as innate immune cells (resident macrophages) of the central nervous system, microglia are one of the causes of brain damage in the nervous system after aSAH (31). When activated, microglia produce numerous proinflammatory cytokines, such as interleukin-1 (IL-1), interleukin-6 (IL-6), and tumor necrosis factor-α (TNF-α), which trigger early neuroinflammatory responses and oxidative stress (32, 33). In addition, astrocytes play an important role in maintaining the integrity of blood-brain barrier and supporting neuronal activity (34) and can differentiate into both proinflammatory (TYPE A1) and anti-inflammatory (TYPE A2) types in response to different stimuli (35). The breakdown products of red blood cells contribute to the transformation of astrocytes into type A1, resulting in increased vascular permeability and impaired the integrity of blood-brain barrier. Under the dual factors of proinflammatory cytokines and increased of blood-brain barrier permeability, the inflammatory response is accelerated, leading to neuronal apoptosis (28).

As an inflammation related protein in the acute phase, C-reactive protein (CRP) is widely used as a marker of inflammation and tissue damage. CRP is produced by the liver. When the body is injured or under the action of inflammatory factors, the liver will rapidly produce CRP and release it into the peripheral blood. The CRP in peripheral blood increased rapidly in a short time (36–38). In patients with aSAH complicated with acute hydrocephalus, a large number of pro-inflammatory cytokines are produced under the inflammatory signal transduction pathway, and the liver rapidly produces a large amount of CRP under the action of IL-1, IL-6 and other factors (39). Similarly, proinflammatory factors promote neutrophil increased recruitment and decreased apoptosis. Neutrophil can release a large number of inflammatory mediators to participate in inflammatory response and pathological nerve injury, resulting in brain edema, blood-brain barrier destruction and secondary brain injury, affecting the prognosis of patients (16, 40, 41).

In this study, it was found that the levels of serum CRP and neutrophil were higher in poor prognosis group 6 months after operation than in good prognosis group, and the increased levels of serum CRP and neutrophil were independent risk factors for poor prognosis 6 months after operation in patients with aSAH complicated with hydrocephalus. However, CRP and neutrophil are non-specific markers of inflammation that can be elevated in any tissue injury. Therefore, combined Hunt-Hess grade can improve its clinical value.

### Hunt-Hess grade

Hunt-Hess grade is an important grade to evaluate the neurological injury and consciousness level of aSAH patients, which can be divided into I-V grade, which directly reflects the severity of the disease to a certain extent. In Hunt-Hess grade I to III patients, early surgery is generally considered necessary because of less bleeding and mild disease. Most patients have a good prognosis after aggressive emergency surgery. The clinical prognosis of Hunt-Hess grade IV–V intracranial aneurysms is poor regardless of surgery or conservative treatment. Most of these patients are treated conservatively, and further surgical treatment is needed after the condition is stable. Many studies have shown that the higher the Hunt-Hess grade in aSAH patients, the higher the risk of poor prognosis (42–44). This study found that the Hunt-Hess grade of patients with aSAH complicated with hydrocephalus at 6 months after surgery in the poor prognosis group was higher than that in the good prognosis group, and higher Hunt-Hess grade at admission was an independent risk factor for poor prognosis in patients with aSAH complicated with hydrocephalus at 6 months after surgery. However, the Hunt-Hess grading evaluation is greatly affected by the patient's own consciousness state and subjective factors of the surgeon, and the combination of biomarkers (such as CRP and neutrophils) can improve the accuracy of prognosis prediction.

### Age

The prognosis of elderly aSAH patients with hydrocephalus is poor. The reason may be that the older the patient is, the worse the basic condition is. In addition, elderly patients are often accompanied by brain parenchyma atrophy, enlargement of the subarachnoid space, and aneurysm rupture of the subarachnoid space to accommodate more blood (45). In addition, the degree of meningeal fibrosis increases with age, resulting in impaired CSF circulation and reduced CSF absorption (18). In the case of aSAH, older people are more likely to develop hydrocephalus, and patients' neurons are more likely to be damaged. Age is an independent risk factor for poor prognosis in aSAH patients with acute hydrocephalus.

In this study, Surgical clipping and higher Hunt-Hess grade were independent risk factors for postoperative DCI in patients with aSAH complicated with acute hydrocephalus. Postoperative DCI is one of the high risk factors for poor prognosis in patients 6 months after surgery. The nomogram was established to assess the probability of postoperative DCI according to the patient 's Hunt-Hess grade at admission and the type of surgery used, thereby proactively taking preventive measures against DCI early. Older age, increased CRP and neutrophil levels, and higher Hunt-Hess grade were independent risk factors for adverse outcome 6 months after operation in patients with aSAH complicated with acute hydrocephalus. The Nomogram was established by combining “age,” “CRP,” “neutrophil” and “Hunt-Hess grade.” The possibility of poor prognosis of patients can be evaluated within 24 h after admission, so that early measures can be taken to improve the prognosis of patients. The advantage of Nomogram as a multi-factor prediction tool is that it can provide quantifiable probabilities and reflect the contribution of each included factor to the prediction. The predicted probability of postoperative DCI and poor prognosis is compared with the actual probability in the calibration curve. The calibration curve showed that the predicted probability of postoperative DCI and poor prognosis was basically consistent with the actual probability. This suggests that nomogram has good clinical application value in this study and can assist in clinical decision making. For patients with high risk of postoperative DCI and poor prognosis, a high risk warning mechanism should be established, disease monitoring should be strengthened, surgical plan should be carefully selected, and intervention measures should be taken as early as possible to reduce the risk of adverse prognosis.

## Conclusion

In conclusion, Hunt-Hess grade at admission and surgical clipping were independent predictors of postoperative DCI. The detection of CRP and neutrophil levels in aSAH patients with hydrocephalus on admission combined with Hunt-Hess grading scale can identify high-risk groups with poor prognosis and provide early intervention. At the same time, it also helps clinicians to make precise treatment, reduce the disability rate and improve the quality of life as much as possible.

## Limitation

The detection of serum CRP and neutrophil levels at admission and the Hunt-Hess grading assessment are simple, rapid and practical, and have great prognostic value for patients with aSAH complicated with acute hydrocephalus 6 months after surgery. However, this study had the following limitations: it was a retrospective, single-center study with a small sample size; In addition, serum samples were collected from all patients within 24 h after admission, but there was no dynamic observation of the changes of serum samples in multiple periods before surgery. For patients with aSAH complicated with acute hydrocephalus before admission, the occurrence of postoperative DCI is associated with poor prognosis. Therefore, in future studies, when these patients developed DCI after surgery, immediately take blood several times in time periods to dynamically observe the changes of indicators. Patients who developed DCI after surgery are studied to investigate the risk factors for poor prognosis 6 months after surgery. In addition, subsequent multicenter prospective clinical trials are needed to further verify these risk factor effect, and subsequent basic experiments are needed to explore its pathophysiological mechanism and understand its role in disease progression, so as to guide clinicians in the treatment and judgment of disease.

## Data availability statement

The original contributions presented in the study are included in the article/supplementary material, further inquiries can be directed to the corresponding authors.

## Ethics statement

Ethical review and approval was not required for the study on human participants in accordance with the local legislation and institutional requirements. Written informed consent for participation was not required for this study in accordance with the national legislation and the institutional requirements.

## Author contributions

Conceptualization and methodology: JG. Software and formal analysis, data analysis and interpretation, and writing—original draft: LW and QZ. Funding acquisition: ZC, JH, and HA-B. Resources: JG, FH, ZZ, YG, ZC, JH, HA-B, GF, and TL. Data curation: LW, QZ, GZ, and WZ. Writing—review and editing: LW, QZ, and JG. Supervision: WC, YW, and XL. All authors contributed to the article and approved the submitted version.

## Funding

This work was supported by Natural Science Foundation of Henan Province, [Grant numbers: 222300420412]; by the Senior Specialist Foreign Expert Project, [Grant numbers: G20190126006]; and by Department of Science and Technology of Henan Province.

## Conflict of interest

The authors declare that the research was conducted in the absence of any commercial or financial relationships that could be construed as a potential conflict of interest.

## Publisher's note

All claims expressed in this article are solely those of the authors and do not necessarily represent those of their affiliated organizations, or those of the publisher, the editors and the reviewers. Any product that may be evaluated in this article, or claim that may be made by its manufacturer, is not guaranteed or endorsed by the publisher.

## Abbreviations

aSAH: aneurysmal arachnoid hemorrhage
AUC: areas under curve
CPe: choroid plexus epithelium
CRP: C-reactive protein
CSF: cerebrospinal fluid
CT: Computed tomography
CTA: CT angiography
DCI: delayed cerebral ischemia
DSA: digital subtraction angiography
IL-1: interleukin 1
IL-6: interleukin 6
IVH: intraventricular hemorrhage
mRS: modified Rankin scale
PHH: post-haemorrhagic hydrocephalus
ROC: receiver operating characteristic
TLR4: Toll-like receptor 4
TNF-α: tumor necrosis factor α.

## References

[1] Etminan N Chang HS Hackenberg K de Rooij NK Vergouwen MDI Rinkel GJE. Worldwide incidence of aneurysmal subarachnoid hemorrhage according to region, time period, blood pressure, and smoking prevalence in the population: a systematic review and meta-analysis. JAMA Neurol. (2019) 76:588–97 10.1001/jamaneurol.2019.0006 30659573 PMC6515606

[2] Macdonald RL Schweizer TA. Spontaneous subarachnoid haemorrhage. Lancet. (2017) 389:655–66. 10.1016/S0140-6736(16)30668-7 27637674

[3] Kundra S Mahendru V Gupta V Choudhary AK. Principles of neuroanesthesia in aneurysmal subarachnoid hemorrhage. J Anaesthesiol Clin Pharmacol. (2014) 30:328–37 10.4103/0970-9185.137261 25190938 PMC4152670

[4] Neifert SN Chapman EK Martini ML Shuman WH Schupper AJ Oermann EK. Aneurysmal subarachnoid hemorrhage: the last decade. Transl Stroke Res. (2021) 12:428–46. 10.1007/s12975-020-00867-0 33078345

[5] Ding C Kang D Chen P Wang Z Lin Y Wang D. Early stage neuroglobin level as a predictor of delayed cerebral ischemia in patients with aneurysmal subarachnoid hemorrhage. Brain Behav. (2020) 10:e01547. 10.1002/brb3.1547 32026621 PMC7066341

[6] Masson A Boulouis G Janot K Herbreteau D Cottier JP Bibi R. Acute hydrocephalus and delayed cerebral infarction after aneurysmal subarachnoid hemorrhage. Acta Neurochir. (2022) 164:2401–8. 10.1007/s00701-022-05321-8 35918615

[7] Petridis AK Kamp MA Cornelius JF Beez T Beseoglu K Turowski B. Aneurysmal subarachnoid hemorrhage. Dtsch Arztebl Int. (2017) 114:226–36 10.3238/arztebl.2017.0226 28434443 PMC5624452

[8] Lu J Ji N Yang Z Zhao X. Prognosis and treatment of acute hydrocephalus following aneurysmal subarachnoid haemorrhage. J Clin Neurosci. (2012) 19:669–72. 10.1016/j.jocn.2011.06.032 22361252

[9] Germanwala AV Huang J Tamargo RJ. Hydrocephalus after aneurysmal subarachnoid hemorrhage. Neurosurg Clin N Am. (2010) 21:263–70 10.1016/j.nec.2009.10.013 20380968

[10] Chen S Luo J Reis C Manaenko A Zhang J. Hydrocephalus after subarachnoid hemorrhage: pathophysiology, diagnosis, and treatment. Biomed Res Int. (2017) 2017:8584753. 10.1155/2017/8584753 28373987 PMC5360938

[11] Hong DY Kim SY Kim JY Kim JW. Red blood cell distribution width is an independent predictor of mortality in patients with aneurysmal subarachnoid hemorrhage. Clin Neurol Neurosurg. (2018) 172:82–6. 10.1016/j.clineuro.2018.06.044 29986200

[12] Turner CL Budohoski K Smith C Hutchinson PJ Kirkpatrick PJ Murray GD. Elevated Baseline C-reactive protein as a predictor of outcome after aneurysmal subarachnoid hemorrhage: data from the simvastatin in aneurysmal subarachnoid hemorrhage (Stash) trial. Neurosurgery. (2015) 77:786–92. 10.1227/NEU.0000000000000963 26280117 PMC4605277

[13] Guresir E Coch C Fimmers R Ilic I Hadjiathanasiou A Kern T. Initial inflammatory response is an independent predictor of unfavorable outcome in patients with good-grade aneurysmal subarachnoid hemorrhage. J Crit Care. (2020) 60:45–9. 10.1016/j.jcrc.2020.07.018 32739759

[14] Zhao B Yang H Zheng K Li Z Xiong Y Tan X. Predictors of good functional outcomes and mortality in patients with severe re-bleeding after aneurysmal subarachnoid hemorrhage. Clin Neurol Neurosurg. (2016) 144:28–32. 10.1016/j.clineuro.2016.02.042 26963087

[15] Okazaki T Hifumi T Kawakita K Shishido H Ogawa D Okauchi M. Blood glucose variability: a strong independent predictor of neurological outcomes in aneurysmal subarachnoid hemorrhage. J Intensive Care Med. (2018) 33:189–95. 10.1177/0885066616669328 27630011

[16] Zhang Y Li L Jia L Li T Di Y Wang P. Neutrophil counts as promising marker for predicting in-hospital mortality in aneurysmal subarachnoid hemorrhage. Stroke. (2021) 52:3266–75. 10.1161/STROKEAHA.120.034024 34167330

[17] Wessell AP Kole MJ Cannarsa G Oliver J Jindal G Miller T. A sustained systemic inflammatory response syndrome is associated with shunt-dependent hydrocephalus after aneurysmal subarachnoid hemorrhage. J Neurosurg. (2018) 130:1–8. 10.3171/2018.1.JNS172925 29957109

[18] Dorai Z Hynan LS Kopitnik TA Samson D. Factors related to hydrocephalus after aneurysmal subarachnoid hemorrhage. Neurosurgery. (2003) 52:763–9. 10.1227/01.NEU.0000053222.74852.2D 12657171

[19] Wilson TJ Stetler WR Jr Davis MC Giles DA Khan A Chaudhary N. Intraventricular hemorrhage is associated with early hydrocephalus, symptomatic vasospasm, and poor outcome in aneurysmal subarachnoid hemorrhage. J Neurol Surg A Cent Eur Neurosurg. (2015) 76:126–32. 10.1055/s-0034-1394189 25545809

[20] Karimy JK Zhang J Kurland DB Theriault BC Duran D Stokum JA. Inflammation-dependent cerebrospinal fluid hypersecretion by the choroid plexus epithelium in post-hemorrhagic hydrocephalus. Nat Med. (2017) 23:997–1003. 10.1038/nm.4361 28692063

[21] Metayer T Orset C Ali C Furon J Szabla N Emery E. Bumetanide lowers acute hydrocephalus in a rat model of subarachnoid hemorrhage. Acta Neurochir. (2022) 164:499–505. 10.1007/s00701-021-05088-4 35094147

[22] Dorhout Mees SM Kerr RS Rinkel GJ Algra A Molyneux AJ. Occurrence and impact of delayed cerebral ischemia after coiling and after clipping in the international subarachnoid aneurysm trial (Isat). J Neurol. (2012) 259:679–83. 10.1007/s00415-011-6243-2 21947244 PMC3319891

[23] de Oliveira JG Beck J Ulrich C Rathert J Raabe A Seifert V. Comparison between clipping and coiling on the incidence of cerebral vasospasm after aneurysmal subarachnoid hemorrhage: a systematic review and meta-analysis. Neurosurg Rev. (2007) 30:22–30. 10.1007/s10143-006-0045-5 17061137

[24] Lucke-Wold BP Logsdon AF Manoranjan B Turner RC McConnell E Vates GE. Aneurysmal subarachnoid hemorrhage and neuroinflammation: a comprehensive review. Int J Mol Sci. (2016) 17:497. 10.3390/ijms17040497 27049383 PMC4848953

[25] Sercombe R Dinh YR Gomis P. Cerebrovascular inflammation following subarachnoid hemorrhage. Jpn J Pharmacol. (2002) 88:227–49. 10.1254/jjp.88.227 11949877

[26] Kuo LT Huang AP. The pathogenesis of hydrocephalus following aneurysmal subarachnoid hemorrhage. Int J Mol Sci. (2021) 22:5050. 10.3390/ijms22095050 34068783 PMC8126203

[27] Kwon MS Woo SK Kurland DB Yoon SH Palmer AF Banerjee U. Methemoglobin is an endogenous toll-like receptor 4 ligand-relevance to subarachnoid hemorrhage. Int J Mol Sci. (2015) 16:5028–46. 10.3390/ijms16035028 25751721 PMC4394463

[28] Wu F Liu Z Li G Zhou L Huang K Wu Z. Inflammation and oxidative stress: potential targets for improving prognosis after subarachnoid hemorrhage. Front Cell Neurosci. (2021) 15:739506. 10.3389/fncel.2021.739506 34630043 PMC8497759

[29] Sarrafzadeh A Schlenk F Gericke C Vajkoczy P. Relevance of cerebral interleukin-6 after aneurysmal subarachnoid hemorrhage. Neurocrit Care. (2010) 13:339–46. 10.1007/s12028-010-9432-4 20725805

[30] Bozza MT Jeney V. Pro-inflammatory actions of heme and other hemoglobin-derived damps. Front Immunol. (2020) 11:1323. 10.3389/fimmu.2020.01323 32695110 PMC7339442

[31] Kim YS Joh TH. Microglia, major player in the brain inflammation: their roles in the pathogenesis of Parkinson's disease. Exp Mol Med. (2006) 38:333–47. 10.1038/emm.2006.40 16953112

[32] Hanafy KA. The role of microglia and the Tlr4 pathway in neuronal apoptosis and vasospasm after subarachnoid hemorrhage. J Neuroinflammation. (2013) 10:83. 10.1186/1742-2094-10-83 23849248 PMC3750560

[33] Lin T Ding L Lin Y Liu C Wang C Wu D. Pharmacological inhibition of Tlr4-Nf-Kappab signaling by Tak-242 attenuates hydrocephalus after intraventricular hemorrhage. Int Immunopharmacol. (2022) 103:108486. 10.1016/j.intimp.2021.108486 34973529

[34] Cunningham C Dunne A Lopez-Rodriguez AB. Astrocytes: heterogeneous and dynamic phenotypes in neurodegeneration and innate immunity. Neuroscientist. (2019) 25:455–74. 10.1177/1073858418809941 30451065 PMC6525076

[35] Xu X Zhang A Zhu Y He W Di W Fang Y. Mfg-E8 reverses microglial-induced neurotoxic astrocyte (A1) Via Nf-Kappab and Pi3k-Akt pathways. J Cell Physiol. (2018) 234:904–14. 10.1002/jcp.26918 30076715

[36] Noh S Kim J Kim G Park C Jang H Lee M. Recent advances in Crp biosensor based on electrical, electrochemical and optical methods. Sensors. (2021) 21:3024. 10.3390/s21093024 33925825 PMC8123455

[37] Muroi C Hugelshofer M Seule M Tastan I Fujioka M Mishima K. Correlation among systemic inflammatory parameter, occurrence of delayed neurological deficits, and outcome after aneurysmal subarachnoid hemorrhage. Neurosurgery. (2013) 72:367–75. 10.1227/NEU.0b013e31828048ce 23208059

[38] Gaastra B Barron P Newitt L Chhugani S Turner C Kirkpatrick P. Crp (C-Reactive Protein) in outcome prediction after subarachnoid hemorrhage and the role of machine learning. Stroke. (2021) 52:3276–85. 10.1161/STROKEAHA.120.030950 34238015

[39] Eklund CM. Proinflammatory cytokines in Crp baseline regulation. Adv Clin Chem. (2009) 48:111–36. 10.1016/S0065-2423(09)48005-3 19803417

[40] Ye ZN Wu LY Liu JP Chen Q Zhang XS Lu Y. Inhibition of leukotriene B4 synthesis protects against early brain injury possibly via reducing the neutrophil-generated inflammatory response and oxidative stress after subarachnoid hemorrhage in rats. Behav Brain Res. (2018) 339:19–27. 10.1016/j.bbr.2017.11.011 29133197

[41] Wei P You C Jin H Chen H Lin B. Correlation between serum Il-1beta levels and cerebral edema extent in a hypertensive intracerebral hemorrhage rat model. Neurol Res. (2014) 36:170–5. 10.1179/1743132813Y.0000000292 24410061

[42] Zhang F Li P Zhang C Wang L Jing SQ. The prognosis factors for endovascular coiling of aneurysm in patients with ruptured intracranial aneurysm. J Craniofac Surg. (2017) 28:e535–e9. 10.1097/SCS.0000000000003818 28692523

[43] Jiang C Luan D Wang C Liu Q Han J Li G. Risk and prognostic factors for rupture of intracranial aneurysms during endovascular embolization. World Neurosurg. (2019) 129:e641–e9. 10.1016/j.wneu.2019.05.233 31158537

[44] Lu C Feng Y Li H Li S Gu L Liu W. Microsurgical treatment of 86 anterior choroidal artery aneurysms: analysis of factors influencing the prognosis. J Neurol Surg A Cent Eur Neurosurg. (2020) 81:501–7. 10.1055/s-0039-3400952 32559770

[45] Jeong TS Yoo CJ Kim WK Yee GT Kim EY Kim MJ. Factors related to the development of shunt-dependent hydrocephalus following subarachnoid hemorrhage in the elderly. Turk Neurosurg. (2018) 28:226–33. 10.5137/1019-5149.JTN.19752-16.1 28497436

